# Poly-ubiquitination in TNFR1-mediated necroptosis

**DOI:** 10.1007/s00018-016-2191-4

**Published:** 2016-04-11

**Authors:** Yves Dondelinger, Maurice Darding, Mathieu J. M. Bertrand, Henning Walczak

**Affiliations:** Inflammation Research Center, VIB, Technologiepark 927, Zwijnaarde, 9052 Ghent, Belgium; Department of Biomedical Molecular Biology, Ghent University, Technologiepark 927, Zwijnaarde, 9052 Ghent, Belgium; Centre for Cell Death, Cancer, and Inflammation (CCCI), UCL Cancer Institute, University College London, London, UK

**Keywords:** Ubiquitination, Necroptosis, TNFR1, RIPK1, c IAP1/2, LUBAC, A20, CYLD

## Abstract

Tumor necrosis factor (TNF) is a master pro-inflammatory cytokine, and inappropriate TNF signaling is implicated in the pathology of many inflammatory diseases. Ligation of TNF to its receptor TNFR1 induces the transient formation of a primary membrane-bound signaling complex, known as complex I, that drives expression of pro-survival genes. Defective complex I activation results in induction of cell death, in the form of apoptosis or necroptosis. This switch occurs via internalization of complex I components and assembly and activation of secondary cytoplasmic death complexes, respectively known as complex II and necrosome. In this review, we discuss the crucial regulatory functions of ubiquitination—a post-translational protein modification consisting of the covalent attachment of ubiquitin, and multiples thereof, to target proteins—to the various steps of TNFR1 signaling leading to necroptosis.

## Introduction

Host defense against invading microbes is mediated by the selective sensing of some of their conserved components by an array of innate immune receptors that are mainly expressed by cells of the immune system and barrier cells that line the outside world. Recognition of these so-called pathogen-associated molecular patterns (PAMPs) by the pattern recognition receptors (PRRs) leads to the activation of signaling pathways [including the mitogen-activated protein kinase (MAPK) and nuclear factor-κB (NF-κB) pathways] that collectively drive the production and release of pro-inflammatory cytokines and chemokines [1]. These cytokines can modulate the innate immune response by binding to different plasma membrane receptors, including members of the tumor necrosis factor receptor superfamily (TNFR-SF). In addition to their ability to further induce inflammatory mediators, some TNFR-SF members known as death receptors (DRs) possess a cytoplasmic death domain (DD) that allows them to transduce a regulated pro-death signal resulting in apoptotic or necroptotic death of the cell [2]. The combination of inflammatory cytokine production and activation of cell death pathways alerts the immune system and clears the potentially harmful microbes from the organism. Although crucial for the protection of the organism from microbial insult, the inflammation response needs tight regulation because inappropriate inflammatory signals and excessive cell death are at the origin of various pathologies.

Ubiquitination, a post-translational modification of proteins consisting in the covalent attachment of the small 8 kDa protein ubiquitin (Ub), and multiples thereof, to target proteins, plays a crucial role in the regulation of various aspects of the inflammatory response [3]. Ubiquitination is a dynamic process that is catalyzed by the action of a three-step enzymatic cascade involving a Ub-activating enzyme (E1), a Ub-conjugating enzyme (E2) and a Ub-ligase (E3), and which is negatively regulated by de-ubiquitinases (DUBs). Ubiquitin is first loaded on the E1 in an ATP-dependent manner. Next, Ub is discharged from the E1 and transferred to the E2. The E2 can then bind to an E3 that mediates the transfer of Ub to a lysine (K) residue of a substrate via an isopeptide bond. The attachment of a single moiety of Ub to a target protein is referred to as mono-ubiquitination. Since Ub itself contains seven lysine residues (K6, K11, K27, K29, K33, K48 and K63) that can all serve as acceptor sites for another Ub molecule, ubiquitination can also result in the conjugation of at least seven different poly-Ub chains to a substrate. An additional chain, known as the M1-linked poly-Ub chain (also called linear Ub chain) can be generated via a peptide bond between the N-terminal methionine (M1) of one Ub to the C-terminal glycine of another [4–6]. The Linear UBiquitin chain Assembly Complex (LUBAC) is the only E3 identified so far capable of exclusively generating these M1-linked ubiquitin linkages. Because the internal lysines of Ub are present in different positions on the surface of the protein, each of the eight possible Ub linkages adopts a structurally distinct conformation [7]. Proteins harboring Ub-binding domains (UBDs) specifically recognize these structurally different Ub linkages, and thereby translate the Ub code into distinct cellular functions [8]. De-ubiquitinases terminate or modulate the Ub-dependent signal by removing the Ub moieties from the substrates. Two major classes of DUBs exist: substrate-specific DUBs and linkage-specific DUBs [9]. The specificity of the former class is regulated by substrate recognition and the enzymes that constitute that group are usually able to cleave any type of linkage. Most members of the Ub-specific protease (USP) family are considered substrate-specific DUBs. In contrast, the other families of DUBs are mainly linkage-specific and are able to process only few specific types of Ub linkages. For both DUB types, substrate specificity can be further regulated by the presence of additional UBDs, the binding to UBD-containing adaptor proteins and post-translational modifications [9].

It is evident that the spatiotemporal regulation of the expression and/or activity of all these different types of Ub-modulating enzymes tightly controls the intracellular Ub code and, as a consequence, also the cellular response to a specific trigger. Indeed, interfering with E3s and DUBs greatly affects the responses activated downstream of several innate immune receptors. In this review, we focus on the TNF signaling pathway and discuss the literature on the role of ubiquitination in the regulation of TNF-mediated necroptosis, a caspase-independent regulated form of necrosis. During this cell death process, and in contrast to apoptosis, the cell and its organelles swell until this process finally culminates in plasma membrane rupture and cell death. TNF-mediated necroptosis relies on RIPK1 kinase-dependent assembly of a cytosolic death complex, known as the necrosome, whose core components apart from RIPK1 are RIPK3 and MLKL [10–13]. The necrosome presumably originates from the dissociation of a primary plasma membrane-associated signaling complex, called TNFR1 complex I or TNRF1-SC, which forms within seconds following engagement of TNFR1 by TNF [14, 15]. It is therefore important to start our review by describing the role of ubiquitination in the regulation of TNFR1 complex I assembly and function.

## Role of poly-ubiquitination in regulating TNFR1 complex I assembly

**Fig. 1:**
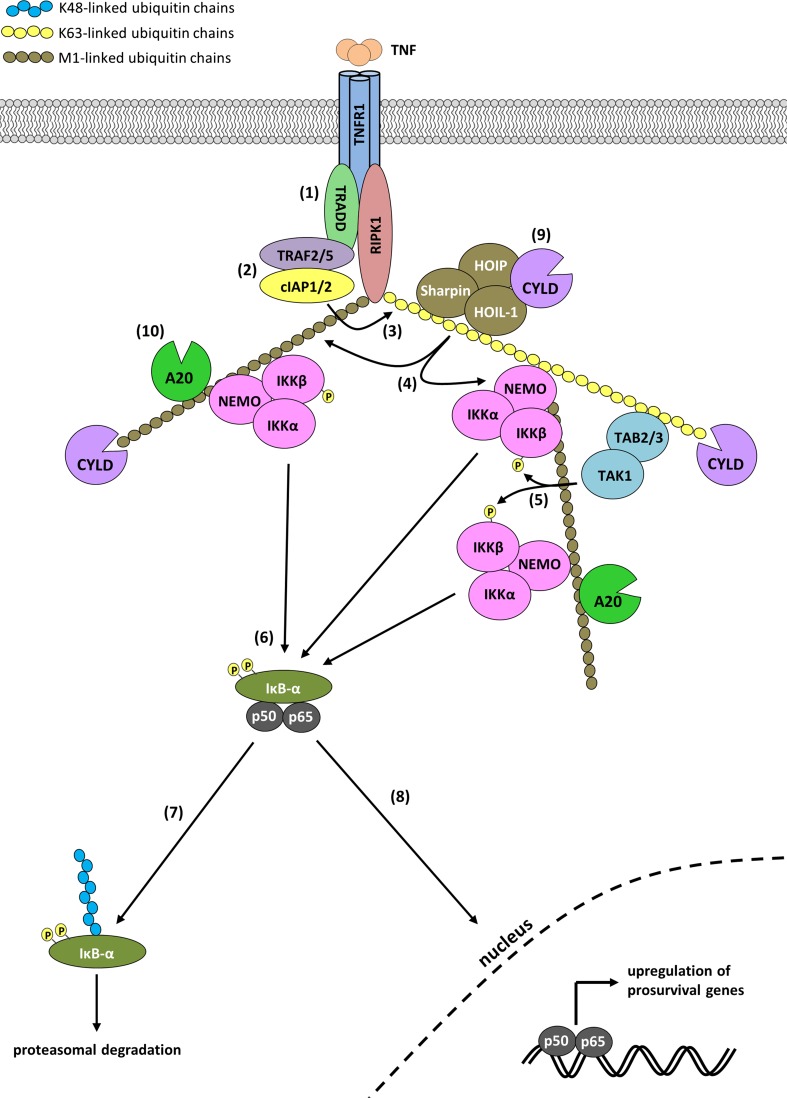
TNFR1 complex I assembly and NF-κB activation. TNF ligation to trimeric TNFR1 leads to recruitment of TRADD and RIPK1 (*1*). TRAF2/5 and cIAP1/2 are then recruited to TRADD (*2*), which allows cIAP1/2 to conjugate RIPK1 with Ub chains, including K63-linked chains (*3*). The Ub chains added to RIPK1 then allow further recruitment of the TAK1, IKK and LUBAC complexes via the respective Ub binding domains of TAB 2/3, NEMO and HOIL/Sharpin. Once recruited, LUBAC adds M1-linked Ub chains to several complex I components, including RIPK1 and NEMO. This leads to the recruitment of additional IKK complexes (*4*). Activated TAK1 then activates IKKα/IKKβ by phosphorylation (*5*). IKKβ subsequently phosphorylates IκBα at Ser32 and Ser36 (*6*) thereby marking it for K48-linked ubiquitination and subsequent proteasomal degradation (*7*). Released from IκBα inhibition, the p50/p65 NF-κB transcription factor translocates to the nucleus to induce expression of several pro-survival genes (*8*). Ubiquitination in TNFR1 complex I is negatively regulated by LUBAC-recruited CYLD, which removes both K63- and M1-linked Ub chains from several substrates, including RIPK1 (*9*). In contrast, binding of A20 to M1-linked chains stabilizes them by preventing their degradation (*10*)

Binding of TNF to TNFR1 induces the independent recruitment of the adaptor protein TRADD and the kinase RIPK1 to the receptor’s DD via homotypic DD interactions (Fig. 1). Complex I-recruited TRADD serves as platform for recruitment of TRAF2 and/or TRAF5. The cIAP interaction motif (CIM) contained within TRAF2 allows recruitment of two closely related members of the Inhibitor of Apoptosis Protein (IAP) family, the Ub E3s cIAP1 and cIAP2 [16, 17]. Following their recruitment, cIAP1/2 ubiquitinate components of complex I, such as RIPK1 and cIAP1 itself, with K63-, K11- and K48-linked poly-Ub chains [18–23]. The Ub chains generated by cIAP1/2 in turn allow the subsequent recruitment of LUBAC, a Ub E3 complex consisting of the central catalytic component HOIP (RNF31), HOIL-1 (RBCK1) and SHARPIN (SIPL1) that exclusively generates M1-linked poly-Ub chains [17, 23–26]. LUBAC adds M1-linked Ub chains to components of complex I, including RIPK1, NEMO, TRADD and TNFR1 itself [17, 23, 26–28]. The Ub chains placed on components of complex I by cIAP1/2 and LUBAC serve as scaffold for recruitment and retention of the TAB 2/3/TAB 1/TAK1 and NEMO/IKKα/IKKβ kinase complexes (Fig. 1). Recruitment of these complexes to the Ub chains is mediated by the UBDs present in TAB 2/3 and NEMO, respectively [29–32]. Whereas TAB 2/3 specifically binds K63-linked poly-Ub chains, NEMO binds to M1-, K63-, and K11-linked chains, yet with approximately 100-fold higher affinity to M1-linked over K63- and K11-linked chains [22, 30, 32–35]. Following activation, TAK1 activates the downstream MAPKs (JNK, p38 and ERK) and IKKβ by phosphorylation. IKKβ in turn phosphorylates IκBα, which leads to its K48-linked poly-ubiquitination by the Skp1-Cullin-F-box (SCF)/β-TRCP E3 complex and results in proteasomal degradation of IκBα [36–39]. This liberates the p50/RelA NF-κB dimer from IκBα-imposed inhibition, and allows p50/RelA to translocate to the nucleus where it drives expression of NF-κB target genes (Fig. 1). The Ub-dependent signaling emerging from complex I is negatively regulated by DUBs that mediate disassembly of complex I by hydrolyzing the Ub modifications present in the complex. Some DUBs, such as A20 and Cezanne, are upregulated by TNF-induced NF-κB and reported to be part of a negative feed-back mechanism aimed at repressing NF-κB activation [40, 41]. A20 was initially proposed to repress RIPK1-mediated NF-κB activation by functioning as a DUB removing the K63-linked Ub chains from RIPK1, and as an E3 conjugating RIPK1 with K48-linked Ub chains promoting its proteasomal degradation [41]. Several findings are now questioning the validity of this model, such as the linkage specificity of its DUB activity and, more importantly, the fact that mice harboring mutations affecting A20′s DUB or E3 activities do not develop any signs of inflammation and are grossly normal [42–45]. Other DUBs, such as CYLD and USP21, are constitutively expressed and also implicated in regulating TNFR1 signaling [46–49]. It was recently demonstrated that CYLD is recruited to complex I via direct binding to HOIP independently of LUBAC activity, and that A20 directly binds to M1-linked Ub chains in complex I, therefore requiring the E3 activity of LUBAC for recruitment [28]. Once recruited, CYLD limits NF-κB activation by removing K63-linked and M1-linked poly-Ub chains on several components of complex I, including RIPK1 [28, 47–50]. Interestingly, CYLD and A20 seem to have opposing effect on M1-linked poly-Ub chain stability. Whilst CYLD degrades them, A20 binds to them, thereby preventing their cleavage and, hence, removal (Fig. 1) [28]. Although the specific roles of all the individual DUBs in complex I-dependent signaling are not yet fully understood, the targeted recruitment of individual DUBs to particular linkage types, together with their specificity in hydrolyzing specific Ub linkages, likely serves to fine-tune the precise extent and duration of signaling.

## Role of poly-ubiquitination in regulating the two TNFR1 complex I-dependent cell death checkpoints

TNF-induced necroptosis is not the default cell death pathway activated by TNFR1. Indeed, necroptosis only occurs when caspase-8 activation fails or is inhibited, indicating that caspase-8-mediated apoptosis actively represses necroptosis [51–55]. Since any condition that sensitizes to TNF-induced apoptosis will therefore also sensitize to TNF-induced necroptosis when caspase-8 is additionally inhibited, understanding how ubiquitination regulates caspase-8 activation is therefore of great relevance to comprehend TNF-induced necroptosis.

**Fig. 2:**
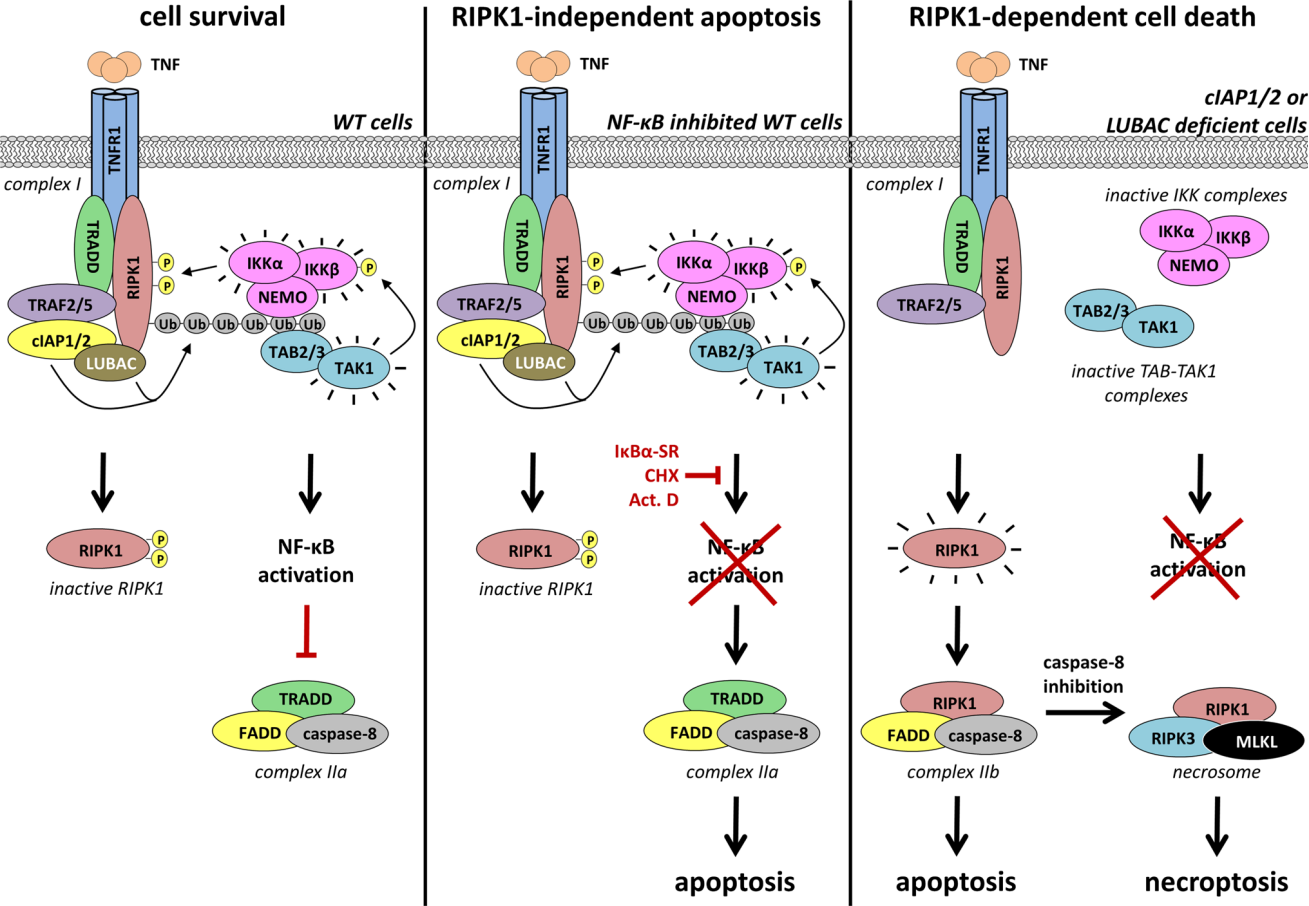
Regulation of the different TNFR1 cell death checkpoints by poly-ubiquitination. *Left panel* cIAP1/2- and LUBAC-mediated ubiquitination of TNFR1 complex I components activates the IKK complex. Active IKKα/β then promote cell survival by NF-κB-dependent upregulation of pro-survival genes that counteract the activation of the cytosolic apoptosis-inducing complex IIa (TRADD-FADD-caspase-8). In addition, IKKα/β directly phosphorylate RIPK1 in complex I thereby preventing RIPK1 activation and, as a consequence, RIPK1-mediated cell death (apoptosis and necroptosis). *Middle panel* inhibition of the NF-κB pathway downstream of the IKK complex, i.e. by an undegradable IκBα (IκBα-SR), the transcription inhibitor actinomycin D (Act. D) or the translation inhibitor cycloheximide (CHX), induces complex IIa-dependent apoptosis. In this scenario, the active IKK complex still prevents RIPK1 activation and RIPK1-mediated cell death (apoptosis and necroptosis). *Right panel* cIAP1/2 or LUBAC deficiency impairs TNF-induced activation of the IKK complex. As a result, the IKK-mediated brake on RIPK1 is relieved. Active RIPK1 then promotes assembly of complex IIb (RIPK1-FADD-caspase-8) resulting in RIPK1-dependent apoptosis. Inhibition of caspase-8 shifts the cell death modality from RIPK1-dependent apoptosis to RIPK1-dependent necroptosis, induced by the necrosome (RIPK1/RIPK3/MLKL)

In most cases, TNFR1 engagement is insufficient to kill cells but instead results in the ubiquitination-dependent activation of gene induction through the NF-κB and MAPK pathways (Fig. 2). However, when the NF-κB-dependent response is inhibited, such as upon genetic deletion of NF-κB, expression of a dominant-negative form of IκBα or in the presence of transcriptional (actinomycin D) or translational (cycloheximide) inhibitors, cells succumb to TNFR1 activation by TNF [56, 57]. Under these circumstances, the switch from a pro-survival to a pro-death response involves the formation of a cytoplasmic caspase-8-activating complex, known as TNFR1 complex II (Fig. 2) [58, 59]. Complex II assembles when TRADD dissociates from complex I and engages FADD in the cytosol [14]. FADD in turn serves as a platform for the recruitment and activation of caspase-8, resulting in apoptotic cell death [60]. The transient formation of complex II normally does not result in cell death since complex I–dependent transcriptional upregulation of pro-survival genes such as FLIP counteracts death induction from complex II. FLIP is highly homologous to caspase-8 but lacks catalytic activity and competes with caspase-8 for recruitment to FADD in TNF complex II, thereby preventing full caspase-8 activation.

It has now become clear that the NF-κB-dependent induction of pro-survival genes is not the only cell death checkpoint regulated by complex I, and that the role of ubiquitination in preventing TNF-mediated cell death exceeds canonical NF-κB activation [61]. Indeed, when ubiquitination events in complex I are perturbed by the absence of the E3 ligases cIAP1/2 or LUBAC, cells also die after TNF stimulation due to increased formation of complex II [19, 58, 62–66]. In this case, complex II formation is highly dependent on RIPK1 and its kinase activity. It is thought that the absence of cIAP1/2 or LUBAC results in insufficient ubiquitination of RIPK1 in complex I, which results in RIPK1 promoting the formation of complex II and cell death. (Fig. 2) [59]. In contrast with complex II-mediated apoptosis induced by inhibiting the NF-kB response downstream, which occurs independently of RIPK1, the kinase activity of RIPK1 is crucial for complex II assembly and apoptosis induction in the absence of cIAP1/2 or LUBAC [58, 67, 68]. Therefore, to distinguish these two different modes of inducing complex II, the former is also called complex IIa, and the latter complex IIb. In addition to apoptosis, deficiency in cIAP1/2 or LUBAC also sensitizes cells to TNF-induced necroptosis that is dependent on the kinase activity of RIPK1 [11, 23, 64, 69, 70]. These findings indicated that cIAP1/2 and LUBAC negatively regulate the pro-death function of RIPK1 on top of promoting canonical NF-κB activation. The Ub chains conjugated to RIPK1 by cIAP1/2 and LUBAC are therefore not only required to activate the canonical NF-κB pathway but also to repress RIPK1 kinase-dependent death (Fig. 2). This concept is supported by the fact that cells expressing a form of RIPK1 that is mutated for its Ub acceptor site (K377R) undergo RIPK1-dependent death following TNFR1 engagement by TNF [31, 71]. Further supporting the notion that cIAP1/2 regulate the pro-death function of RIPK1 directly is the formation of the ‘ripoptosome’, a RIPK1-dependent caspase-8-activating complex similar to complex II. However, in contrast to complex II, it forms spontaneously (independently from death receptors) when cIAP1/2 are depleted [72, 73].

The importance of cIAP1/2- and LUBAC-mediated ubiquitination in preventing uncontrolled RIPK1 activation and consequent cell death has also been elegantly demonstrated in a number of genetic mouse models. While the genetic deletion of the catalytic component of LUBAC, HOIP, is embryonically lethal at day E10.5, a null mutation in the *Sharpin* gene is not lethal but instead results in the development of a severe multi-organ inflammatory phenotype called chronic proliferative dermatitis (*cpdm*) [64, 74, 75]. These *cpdm* mice display severe inflammation in the skin, liver, gut, lung and oesophagus, together with a loss of Peyer’s patches and splenomegaly [74, 76]. The inflammatory phenotype of *cpdm* mice is driven by aberrant TNFR1-mediated cell death [23, 65, 77]. Interestingly, the *cpdm* phenotype can also be completely prevented by crossing with RIPK1 kinase-dead knockin mice as well as with the combination of RIPK3 deficiency and caspase-8 heterozygosity [65, 66]. These genetic studies confirm the role of LUBAC-mediated ubiquitination in repressing RIPK1 pro-death function and demonstrate that in the absence of fully functional LUBAC, the kinase activity of RIPK1 can both induce apoptosis and necroptosis in vivo. The role of cIAP1/2 in vivo has been more difficult to study as the single knock-outs do not show any overt phenotype whereas the double knock-out, similar to the HOIP^−/−^ mice, causes embryonic lethality at day E10.5 due to cardiovascular failure as a consequence of TNFR1-driven yolk sac endothelial cell death [63, 78, 79]. Dysregulation of RIPK1 in these knockouts has also been shown genetically since deletion of RIPK1 slightly delays the lethality of cIAP1^−/−^ cIAP2^−/−^ animals [63]. However, RIPK1 deficiency on its own causes early postnatal lethality by uncontrolled caspase-8-mediated apoptosis and RIPK3-mediated necroptosis [80–82]. It would therefore be interesting to test whether replacing wild-type RIPK1 by a kinase-dead RIPK1 via gene knockin could prevent embryonic lethality of cIAP1/2 or HOIP deficiency as this would impair RIPK1’s pro-death function whilst keeping its pro-survival scaffold function intact.

Importantly, CYLD repression was shown to protect cells from TNF-mediated RIPK1 kinase-dependent apoptosis and necroptosis, which additionally supports the notion that the K63- and M1-linked poly-Ub chains added to complex I components by cIAP1/2 and LUBAC prevent RIPK1 from initiating the formation of the cytosolic death complex II [28, 50, 58, 67, 70]. Apart from functioning as an inhibitor of the canonical NF-κB pathway, A20 is also known as a potent inhibitor of TNF-induced apoptosis and necroptosis, but the mechanism accounting for this function had remained unclear [69, 83, 84]. The recent finding that A20 protects M1-linked poly-Ub chains from CYLD-mediated degradation in complex I now provides a crucial element in our understanding of the cell-protective function of A20 [28].

It was initially believed that the Ub chains conjugated to RIPK1 directly prevent RIPK1 from integrating into complex IIb or the necrosome. More recent findings also suggest that not only the Ub chains themselves, but also the kinases recruited to them control RIPK1 kinase-dependent death. Indeed, IKKα/IKKβ were shown to prevent RIPK1 from integrating into complex IIb through direct phosphorylation in complex I, thereby suggesting a model according to which IKKα/IKKβ directly repress RIPK1 kinase activity (Fig. 2) [85]. In accordance with this model, any conditions affecting IKKα/IKKβ activation downstream of RIPK1 ubiquitination, such as deficiency in TAK1, NEMO or IKKα/IKKβ, were shown to sensitize cells to RIPK1 kinase-dependent apoptosis and necroptosis without affecting RIPK1 ubiquitination in complex I [67, 85–89]. Importantly, this role of IKKα/IKKβ was demonstrated to be independent of NF-κB activation.

## Direct control of the necrosome by poly-ubiquitination

Necroptosis induction was initially reported to occur following recruitment of RIPK3 to a RIPK1/FADD/caspase-8 complex, suggesting that the necrosome originates from an inactive complex IIb. More recent papers however suggested a model in which an independent RIPK1/RIPK3/MLKL complex is formed in parallel to complex IIb [12]. The precise relation between complex IIb and the necrosome is therefore currently unclear and additional work is required to better define whether they represent two independent complexes or variations of the same complex.

**Fig. 3:**
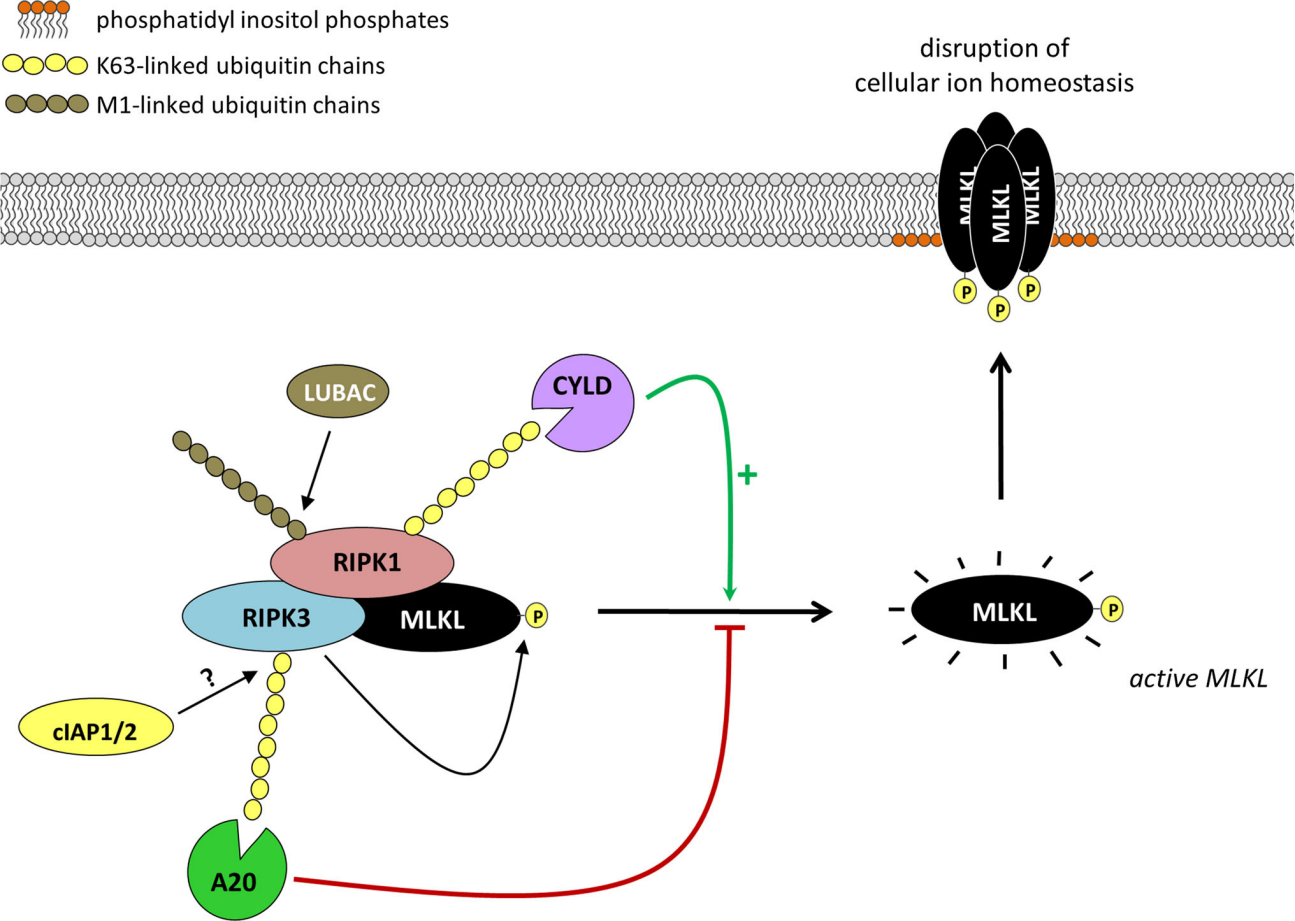
Direct control of the necrosome by poly-ubiquitination. Activated RIPK3 in the necrosome induces MLKL activation by phosphorylation. Active MLKL then translocates to phosphatidyl inositol phosphates in the plasma membrane. Plasma-membrane associated MLKL oligomerizes into high-molecular weight complexes and dysregulates the cellular ion homeostasis resulting in cell swelling and plasma membrane rupture. Although the functional relevance is not entirely clear at this time, RIPK1 and RIPK3 are reported to be poly-ubiquitinated in the necrosome, presumably independently of ubiquitination in TNFR1 complex I. RIPK1 is associated with both M1-linked and K63-linked ubiquitin chains while only K63-linked Ub chains have been described for RIPK3. LUBAC and cIAP1/2 have been involved in the conjugation of these Ub chains. From the DUB point of view, CYLD is reported to promote necroptosis induction by removing the K63-linked Ub chains on RIPK1. In contrast, A20 prevents necroptosis induction by removing the K63-linked Ub chains on RIPK3

As mentioned in the previous section, poly-ubiquitination plays crucial roles in regulating necroptosis by allowing complex I assembly and fine-tuning the two complex-I-dependent cell death check points, namely the NF-κB-dependent induction of pro-survival factors and the NF-κB-independent contribution of RIPK1 to the cell demise. Recent studies suggest that poly-ubiquitination also plays a regulatory role at the level of the cell death-inducing complex itself (Fig. 3). Indeed, TNFR1-induced complex IIb/necrosome-associated components have been reported to show post-translational modifications reminiscent of ubiquitination [67, 90–93]. Necrosome-associated RIPK3 is conjugated with K63-linked poly Ub chains [91], whereas RIPK1 has been reported to contain both M1- and K63-linked Ub chains [90, 93]. It is currently unclear whether these ubiquitin chains regulate the cell death outcome in a positive or negative manner, and whether they additionally regulate a non-cell death signaling pathway emanating from this complex. It appears that only a fraction of the complex IIb/necrosome-associated RIPK1 (and RIPK3) is ubiquitinated, whereas all of the RIPK1 associated with TNFR1 complex I is ubiquitinated [67]. This difference in the amount of ubiquitinated RIPK1 molecules in both complexes may question the importance of these post-translational modifications in the regulation of the cell death complex itself. Indeed, it was demonstrated that HOIP knockdown partially affects RIPK1 ubiquitination in the necrosome, while not affecting necroptotic cell death significantly [93]. The identity of the E3s ubiquitinating the different components of the necrosome are still under debate, and it is not entirely clear whether the poly-Ub chains conjugated to RIPK1 originate from its ubiquitination in TNFR1 complex I. RIPK1 ubiquitination in the necrosome was reported to occur independently of cIAP1/2 and only partially depending on HOIP, which would suggest implication of additional E3s [93]. In vitro studies have shown that cIAP1/2 are able to add different types of poly-Ub chains to RIPK3, yet the relevance of this observation for necroptotic signaling is not established [94]. TRAF2 was also shown to bind to MLKL in cells and was proposed to suppress TNF-induced necroptosis by preventing MLKL to exert its pro-death function [95]. Nevertheless, although MLKL was reported to be ubiquitinated in cells, the suppressive function of TRAF2 appears to occur independently of its supposed activity as a Ub E3 [95, 96].

Poly-ubiquitination at the level of the necrosome was also demonstrated in studies making use of cells depleted of the DUBs CYLD and A20. CYLD deficiency greatly protects against TNF-induced necroptosis and this protection correlates with an increase in RIPK1 poly-ubiquitination [28, 53, 70, 90]. Indeed, CYLD can hydrolyze M1- and K63-linked Ub chains conjugated to RIPK1 [28, 47–50]. Interestingly, Chan and colleagues showed that CYLD-deficiency also affects ubiquitination of RIPK1 in the necrosome, which implies a role of CYLD in arming RIPK1 in both, complex I and the necrosome [90]. In contrast to CYLD, A20-deficient cells are strongly sensitized to TNF-induced necroptosis and a recent study associates this sensitization to increased RIPK3 poly-ubiquitination in the necrosome [91]. In support of this interpretation, the authors showed that mutation of Lys5 in RIPK3 partially inhibited necrosome-associated RIPK3 poly-ubiquitination and TNF-induced necroptotic cell death [91]. It is also of interest to note that absence of A20 does not only increase the amount of poly-ubiquitinated, but also of non-ubiquitinated, RIPK3 in the necrosome. This may be the result of an increased stability of the necrosome in presence of poly-ubiquitinated RIPK3, or reflective of the more upstream role of A20 in the pathway. Indeed, A20 protects M1-linked poly-Ub chains in complex I and thereby prevents RIPK1 from initiating the formation of the cytosolic death-inducing complex II [28].

It is intriguing to note that ubiquitination of RIPK1 and RIPK3 in the necrosome is reported to have opposite effect on necroptosis induction. Those post-translational modifications would repress RIPK1 but activate RIPK3. Future work on the identification of the type of Ub chains conjugated to each protein is clearly needed to provide a molecular basis for the better understanding of these apparent opposing consequences. Also, uncoupling complex I from necrosome assembly may help evaluating the respective contribution of the different E3s and DUBs to the two different steps of the pathway. It might, for example, be interesting to study how the presence or absence of these enzymes affects necroptosis induced by forced homo- and heterodimerization of RIPK1 and RIPK3.

## Concluding remarks

In recent years, great advances have been made in the understanding of the molecular mechanisms of TNFR1-induced necroptosis. However, the exact consequences, functions and interconnections between the diverse post-translational modifications regulating this signaling pathway still remain to be investigated in more detail. Although ubiquitination has clearly emerged as a major regulatory mechanism, the identity of all the substrates, the exact linkage composition of the different poly-Ub chains, the precise roles of the diverse Ub-related enzymes and their specificity for complex I vs. complex II/necrosome still require better understanding. Most of the knowledge acquired over the past years has been generated by the use of cutting-edge biochemistry, often combined with sophisticated in- vivo models employing component-deficient mice and/or cells. It is now important to understand how the activities of the ubiquitination-modifying enzymes are regulated under physiological conditions and how this is deregulated in pathological conditions. It is for example known that binding of TNF to TNFR2 induces degradation of a pool of TRAF2/cIAP1/cIAP2 that consequently affects ubiquitination in TNFR1 complex I and switches the TNFR1-mediated response from survival to death [97]. Co-stimulation with other members of the TNFR superfamily can exert similar effects. What are, however, the conditions that regulate other ubiquitination-modifying enzymes? A possible hint comes from the fact that necroptosis is often associated with the generation of ROS and that the activity of A20 and other OTU deubiquitinases was shown to be regulated by reversible oxidation [98]. Also, viral infection can trigger necroptosis and some viruses have been reported to interfere with the enzymatic activities of endogenous E3s and DUBs [99, 100].

RIPK1 plays a pivotal role in determining the cellular response to TNF. While it functions as a central signaling platform in the TNFR1 complex I to drive NF-κB- and MAPK-mediated cell survival and inflammation, it also plays a crucial role in the cytosolic complex II and, consequently, in the induction of cell death. In accordance, ubiquitination of RIPK1 in both complexes is a key event in regulating the TNFR1 signaling pathway to necroptosis. Nevertheless, necroptosis induced by receptors other than TNFR1, such as TLR3 and DAI, does not require RIPK1. Downstream of these receptors, the RHIM domain required to recruit and activate RIPK3 is respectively provided by TRIF or DAI itself [101–104]. Studying the role of E3s and such as cIAP1/2 and LUBAC as well as DUBs such as A20 and CYLD in the ubiquitination-dependent regulation of necroptosis downstream of these receptors are therefore interesting perspectives.
